# Supplementary material for: Integrating life cycle assessment into supply chain optimization
Source: PLoS One. 2025 Jan 15;20(1):e0316710. doi: 10.1371/journal.pone.0316710 (PMC11734959; doi:10.1371/journal.pone.0316710)
Supplement: S1 File — (PDF) [file pone.0316710.s001.pdf]

Supplementary material for "Integrating life cycle assessment into supply chain optimization"

Selin Hülagü[1*], Wout Dullaert[1◑], A. Sena Eruguz[1◑], Reinout Heijungs[1◑], Dirk Inghels[1◑],

1 Department of Operations Analytics, School of Business and Economics, Vrije Universiteit Amsterdam, Amsterdam, The Netherlands

◑These authors contributed equally to this work.
* s.h.huelague@vu.nl

1 Illustrative example of life cycle assessment

We demonstrate the computational structure of LCA using a hypothetical example of a food supply chain illustrated in Fig 1(a). In this supply chain, there are 3 potential suppliers (S1, S2, and S3), 2 potential plants (P1 and P2), a single customer/market location (C1), and a single type of truck (T1). The LCA methodology is usually performed separately for each possible combination of supplier(s), plant(s), and routing decisions because LCA typically analyzes a well-defined configuration. In this example, with 3 potential suppliers and 2 potential plants, there are 21 possible supplier-plant combinations. In addition, the amount of products/services flowing between each supplier-plant and plant-customer combination must be determined. In the following, we consider a single, specific configuration for the LCA, where the raw material is sourced from supplier S2, produced at plant P2, and distributed to customer C1 by truck T1.

Fig 1(b) shows the typical LCA representation of this supply chain, namely the process-product flow diagram. In this diagram, rectangles represent processes and straight arrows represent product flows. We also indicate environmental flows in the process-product flow diagram with dashed arrows. Fig 1(c) shows the technology matrix $\mathbf{A}$ and the intervention matrix $\mathbf{B^{env}}$ for this example. For example, the second column of the matrices $\mathbf{A}$ and $\mathbf{B^{env}}$ corresponds to the process 'Final product production at plant P2'. According to the coefficients, the production of the final product requires 3 kg of raw material and 20 kgkm of transport from the supplier as inputs. This process produces 1 unit of final product and 25 kg of CO_2 as outputs. Note that the coefficients in $\mathbf{A}$ and $\mathbf{B^{env}}$ are not the actual amounts; they only represent production recipes (e.g. per unit of output).

The FU in this example can be formulated as "5% of an adult woman's daily food energy requirement". In this example, the reference flow (the customer's desired energy requirement) is "100 kcal of energy".

From Eq (1), the column vector $\mathbf{s}$ to satisfy demand is calculated as [1.8 3.0 0.2 0.7].

$$\mathbf{s} = \mathbf{A}^{-1}\mathbf{f} \tag{1}$$

The life cycle inventory vector $\mathbf{g^{env}}$=[99 18 -2], the impact vector $\mathbf{h^{env}}$=[549 2], and the weighted environmental index W^{env}=2749 are determined using Eq (2)-(4).

$$\mathbf{g^{env}} = \mathbf{B^{env}}\mathbf{s} \tag{2}$$

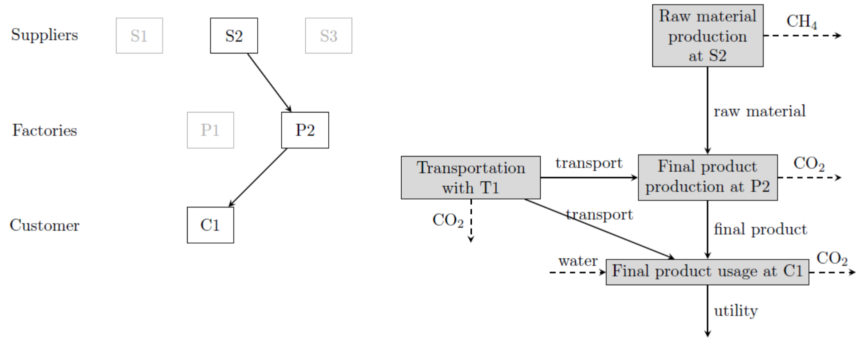

(a) (b)

A =

	Raw material production at S2	Final product production at P2	Product usage at C1	Transportation with T1
raw material [kg]	5	-3	0	0
final product [unit]	0	1	-15	0
transport [kgkm]	0	-20	-50	100
utility [kcal]	0	0	500	0

f =

	Demand
raw material [kg]	0
final product [unit]	0
transport [kgkm]	0
utility [kcal]	100

$\mathbf{B^{env}}$ =

CO_2 [kg]	0	25	15	30
CH_4 [kg]	10	0	0	0
water [m^3]	0	0	-10	0

(c)

$\mathbf{Q^{env}}$ =

	CO_2	CH_4	water
climate change [kg/CO_2]	1	25	0
water use [m^3]	0	0	-1

$\mathbf{w^{env}}$ =

climate change [kg/CO_2]	water use [m^3]
5	2

(d)

Fig 1. Illustration of the computational structure of LCA for a hypothetical supply chain network: (a) supply chain network, (b) process-product flow diagram of products in the supply chain, (c) associated technology matrix **A**, intervention matrix $\mathbf{B^{env}}$, and demand vector **f** of the system, (d) characterization matrix $\mathbf{Q^{env}}$, and weighting vector $\mathbf{w^{env}}$ for the impact assessment.

$$\mathbf{h^{env}} = \mathbf{Q^{env}} \mathbf{g^{env}} \tag{3}$$

$$W^{\text{env}} = \mathbf{w^{env}} \mathbf{h^{env}} \tag{4}$$

As the linear scaling principle of LCA implies, doubling the FU to “10% of an adult woman’s daily food energy requirement” (thus doubling the reference flow to 200 kcal) will double **s**, $\mathbf{g^{env}}$, $\mathbf{h^{env}}$, and W^{env} (e.g. new W^{env}= 5498).

2 Supplementary material for the methodology

In this section, we provide supplementary material on the methodology. In Table 1 we provide the nomenclature for the SCLCO model to assist the reader, and in Table 2 we list frequently used abbreviations throughout the paper. In Section 2.1, we demonstrate the flexibility of SCLCO to accommodate case-specific features by incorporating the requirements of the case study.

Table 1. Symbols frequently used in the SCLCO model.

Symbol	Definition	Dimension
$\mathbf{A}_0$	Technology matrix of the system for strategic process	$N^{\text{str}} \times P^{\text{str}}$
$\mathbf{A}_0^{\mathbf{out}}$	$\mathbf{A}_0$ containing only outflows	$N^{\text{str}} \times P^{\text{str}}$
$\mathbf{A}_t$	Technology matrix of the system for tactical process in time $t = 1, \ldots, T$	$N^{\text{tac}} \times P^{\text{tac}}$
$\mathbf{A}_t^{\mathbf{out}}$	$\mathbf{A}_t$ containing only outflows in time $t = 1, \ldots, T$	$N^{\text{tac}} \times P^{\text{tac}}$
$\mathbf{A'}_t$	Strategic product inflow to the tactical process in time $t = 1, \ldots, T$	$N^{\text{str}} \times P^{\text{tac}}$
$\mathbf{B}_0$	Three pillars intervention matrix for strategic process	$(B + E + J) \times P^{\text{str}}$
$\mathbf{B}_0^{\mathbf{env}}$	Environmental intervention matrix for strategic process	$B \times P^{str}$
$\mathbf{B}_0^{\mathbf{ecn}}$	Economic intervention matrix for strategic process	$E \times P^{str}$
$\mathbf{B}_0^{\mathbf{soc}}$	Social intervention matrix for strategic process	$J \times P^{str}$
$\mathbf{B}_t$	Three pillars intervention matrix for tactical process in time $t = 1, \ldots, T$	$(B + E + J) \times P^{\text{tac}}$
$\mathbf{B}_t^{\mathbf{env}}$	Environmental intervention matrix for tactical process in time $t = 1, \ldots, T$	$B \times P^{tac}$
$\mathbf{B}_t^{\mathbf{ecn}}$	Economic intervention matrix for tactical process in time $t = 1, \ldots, T$	$E \times P^{tac}$
$\mathbf{B}_t^{\mathbf{soc}}$	Social intervention matrix for tactical process in time $t = 1, \ldots, T$	$J \times P^{tac}$
$\mathbf{K}$	Conversion matrix for on-hand stocks and returned products	$N^{\text{tac}} \times N^{\text{tac}}$
$\mathbf{Q}$	Characterization matrix for three pillars of sustainability	$(C + G + L) \times (B + E + J)$
$\mathbf{Q}^{\mathbf{env}}$	Characterization matrix for environmental flows	$C \times B$
$\mathbf{Q}^{\mathbf{ecn}}$	Characterization matrix for economic flows	$G \times E$
$\mathbf{Q}^{\mathbf{soc}}$	Characterization matrix for social flows	$L \times J$
$\mathbf{W}$	Weighting matrix for three pillar of sustainability	$3 \times (C + G + L)$
$\mathbf{e}$	Binary vector for product flows that are not allowed to have a surplus	N^{tac}
$\mathbf{f}_t$	Demand vector for time $t = 1, \ldots, T$	N^{tac}
$\mathbf{s}_0$	Scaling vector for strategic decisions	P^{str}
$\mathbf{s}_t$	Scaling vector for tactical decisions in time $t = 1, \ldots, T$	P^{tac}
$\mathbf{u}_0$	Maximum outflow allowed from strategic processes	N^{str}
$\mathbf{u}_t$	Maximum outflow allowed from tactical processes in time $t = 1, \ldots, T$	N^{tac}
$\mathbf{w}$	Row vector for weighting the three pillars of sustainability	3
$\mathbf{w}^{\mathbf{env}}$	Row vector for weighting environmental impacts	C
$\mathbf{w}^{\mathbf{ecn}}$	Row vector for weighting economic impacts	G
$\mathbf{w}^{\mathbf{soc}}$	Row vector for weighting social impacts	L
B	Number of environmental flows	
C	Number of environmental impact categories	
E	Number of economic flows	
G	Number of economic impact categories	
J	Number of social flows	
L	Number of social impact categories	
N	Number of product flows	
N^{str}	Number of product flows produced by strategic processes	
N^{tac}	Number of product flows produced by tactical processes	
P	Number of processes	
P^{str}	Number of strategic process	
P^{tac}	Number of tactical process	

2.1 Adopting case-specific features

To fully reformulate the case study of [1], we formulate single-objective functions for the three pillars of sustainability and incorporate additional case-specific features (e.g., minimum return fraction, investment budget) into our proposed SCLCO model.

Table 2. Abbreviations frequently used through the paper.

Abbreviation	Definition
CLSC	Closed-Loop Supply Chain
EoL	End-of-Life
ESG	Environmental Social Governance
FU	Functional Unit
ISO	International Organization for Standardization
LCA	Life Cycle Assessment
LCI	Life Cycle Inventory Analysis
LCIA	Life Cycle Impact Assessment
MILP	Mixed-Integer Linear Programming
OR	Operations Research
SCLCO	Supply Chain Life Cycle Optimization
SCO	Supply Chain Optimization
SCM	Supply Chain Management

2.1.1 Objective functions

Environmental and social objectives: The single objective for the environmental pillar ($W^{\mathbf{env}}$) and the social pillar ($W^{\mathbf{soc}}$) presented by [1] can be formulated in SCLCO as Eq (5) and Eq (6).

$$W^{\mathrm{env}} = \sum_{t=0}^{T} \mathbf{w}^{\mathbf{env}} \mathbf{Q}^{\mathbf{env}} \mathbf{B}_t^{\mathbf{env}} \mathbf{s}_t \tag{5}$$

$$W^{\mathrm{soc}} = \sum_{t=0}^{T} \mathbf{w}^{\mathbf{soc}} \mathbf{Q}^{\mathbf{soc}} \mathbf{B}_t^{\mathbf{soc}} \mathbf{s}_t \tag{6}$$

Economic objective: We reformulate the economic objective function presented by [1], which is based on net present value, as Eq (7). Eq (7) includes discounted cash flows at each time period $t = 1, \dots, T$ (first term) taking into account the tax rate, tr, and the interest rate, ir, as well as the cost of capital invested (second term). Two types of strategic economic intervention vectors are used in Eq (7); $\mathbf{b}_0^{\mathbf{ecn1}}$ for the economic flows occurring at each time period $t = 1, \dots, T$ (e.g. driver costs), and $\mathbf{b}_0^{\mathbf{ecn2}}$ for once (e.g. fleet cost). $\mathbf{b}_0^{\mathbf{ecn1}}$ and $\mathbf{b}_0^{\mathbf{ecn2}}$ are P^{str}-dimensional row vectors. The discounted cash flow (8) is determined based on the net earnings at each time period. The net earnings at each time period $t = 1, \dots, T$ (Eq 9) are defined by costs (due to tactical and strategic decisions), earnings, and depreciation of invested capital (due to strategic decisions). Depreciation of the capital invested at each time period $t = 1, \dots, T$ is defined by the vector $\boldsymbol{\gamma}_t \in \mathbb{R}^{P^{\mathrm{str}}}$. For the cash flow at the last time period, the recovery of the salvage value is considered. θ is a $1 \times P^{\mathrm{str}}$-dimensional row vector that contains the percentage salvage value of strategic decisions.

$$W^{\mathrm{ecn}} = \sum_{t=1}^{T} \frac{\mathbf{CF}_t}{(1+ir)^t} + \mathbf{b}_0^{\mathbf{ecn2}} \mathbf{s}_0 \tag{7}$$

$$\mathbf{CF}_t = \begin{cases} \mathbf{NE}_t, & t = 1, 2, ..., T-1 \\ \mathbf{NE}_t - \boldsymbol{\theta} \mathbf{diag}(\mathbf{b}_0^{\mathbf{ecn2}}) \mathbf{s}_0 & t = T \end{cases} \tag{8}$$

$$\mathbf{NE}_t = (1 - tr)(\mathbf{b}_t^{\mathbf{ecn}} \mathbf{s}_t + \mathbf{b}_0^{\mathbf{ecn1}} \mathbf{s}_0) - tr(\mathbf{b}_0^{\mathbf{ecn2}} \mathbf{diag}(\boldsymbol{\gamma}_t) \mathbf{s}_0) \tag{9}$$

2.1.2 Constraints

Tautenhain et al. [1] limit technology selection at the factory level, set a minimum return fraction from customers, limit the amount of investment in trucks, and establish minimum flow requirements. The problem specific requirements can be incorporated into the proposed SCLCO as follows:

Technology selection: Tautenhain et al. [1] limit technology selection to 1 per technology type (production and remanufacturing). To enforce this limitation, we define matrix $\mathbf{G} \in \{0,1\}^{2\times P^{\mathrm{str}}}$. The rows in $\mathbf{G}$ represent technology types (production and remanufacturing), while the columns correspond to strategic processes. A value of 1 in $\mathbf{G}$ indicates a process that contributes to a particular technology type. Constraint (10) ensures that the factory's technology allocation is limited to one. In this constraint, $\mathbf{1}$ denotes the 2-dimensional vector comprised entirely of 1 values.

$$\mathbf{G}\mathbf{s}_0 \leq \mathbf{1} \tag{10}$$

Minimum return fraction: In [1], a minimum of β proportion of the used product from time $t-1$ must be recovered. In the SCLCO framework, we enforce this requirement using matrix $\mathbf{R} \in \{0,1\}^{N^{\mathrm{tac}}\times N^{\mathrm{tac}}}$ and binary vector $\mathbf{e_r} \in \{0,1\}^{N^{\mathrm{tac}}}$. $\mathbf{R}$ conveys the transition from used products to returned products (as a part of matrix $\mathbf{K}$). Vector $\mathbf{e_r}$ includes the value of 1 for the returned products. Constraint (11) ensures that at least β proportion of the used product from time $t-1$ is used as an input in the return flow for recovery at each time period.

$$\mathbf{diag}(\mathbf{e_r})\mathbf{A}_t\mathbf{s}_t + \beta\mathbf{R}\mathbf{A}_{t-1}\mathbf{s}_{t-1} \leq \mathbf{0} \qquad \forall t \in \{2,...,T\} \tag{11}$$

Maximum investment amount in trucks: Tautenhain et al. [1] limit the amount of investment in trucks by *inv*. We introduce constraint (12) to impose such a restriction. In this constraint, $\mathbf{b}_0^{\mathbf{ecn,truck}}$ is the strategic economic intervention vector associated with the truck purchase cost.

$$\mathbf{b}_0^{\mathbf{ecn,truck}}\mathbf{s}_0 \leq inv \tag{12}$$

Mininum inflow/outflow requirement: In the SCO model of [1], strategic decisions establish a minimum requirement for both tactical and strategic decisions. For example, the installation of technology necessitates a minimum level of product production/ remanufacturing, and the purchase of a truck at a location requires the installation of the entity at that location. In SCLCO notation/language, this implies that processes related to strategic decisions impose a lower limit on the product flows associated with both tactical and strategic processes. To impose such lower limits, we introduce the matrix $\mathbf{L}_t$ $t=0,\ldots,T$, $\mathbf{L}''_t$ $t=1,\ldots,T$, and $\mathbf{L}'_t$ $t=1,\ldots,T$. For $t=0$ matrix, $\mathbf{L}_0$ is defined as an $N^{\mathrm{str}} \times P^{\mathrm{str}}$-dimensional matrix. If the existence of a strategic process affects the strategic product outflow, a minimum value is assigned from the column to the row in matrix $\mathbf{L}_0$. For time period $t=1,\ldots,T$, matrix $\mathbf{L}_t$ is an $N^{\mathrm{tac}} \times P^{\mathrm{str}}$-dimensional matrix. If the presence of a strategic process affects the tactical product outflow, a minimum value is assigned from the column to the row in matrix $\mathbf{L}_t$. Matrix $\mathbf{L}''_t$ is an $N^{\mathrm{tac}} \times P^{\mathrm{str}}$-dimensional matrix. If the presence of a strategic process affects the surplus for tactical products, a minimum value is assigned from the column to the row in matrix $\mathbf{L}''_t$. Matrix $\mathbf{L}'_t$ is an $N^{\mathrm{str}} \times P^{\mathrm{tac}}$-dimensional matrix. If existence of a strategic process is affected by the strategic product inflow of a tactical process, a minimum value is assigned from the column to the row in matrix $\mathbf{L}'_t$.

Constraints (13) and (15) enforce the minimum product flow levels based on their interconnection with strategic processes.

$$\mathbf{L}_t\mathbf{s}_0 - \mathbf{A}_t^{\mathbf{out}}\mathbf{s}_t \leq \mathbf{0} \qquad \forall t \in \{0, ..., T\} \tag{13}$$

$$\mathbf{L''}_t\mathbf{s}_0 - \mathbf{K}\mathbf{A}_t\mathbf{s}_t \leq \mathbf{0} \qquad \forall t \in \{1, ..., T\} \tag{14}$$

$$\mathbf{L}'_t\mathbf{s}_0 + \mathbf{A}'_t\mathbf{s}_\mathbf{t} \leq \mathbf{0} \qquad \forall t \in \{1, ..., T\} \tag{15}$$

3 Data for the computational experiments

3.1 Additional data for the case study

We completed the dataset presented in [1] for the following parameters: the fixed workers per technology, transportation costs for air and sea transport, distance between entities, handling costs at hub terminals, environmental impacts of suppliers, and working condition indexes for entities.

Fixed workers per technology is 10 for all technologies: TCH1, TCH2, TCH3, TCH4, TCH5, TCH6.

The transportation cost per kgkm is 0.04 for air transport and 0.01 for sea transport.

The distance between entities, handling costs at hub terminals, working condition indexes for entities, and environmental impacts of suppliers are shown in Table 3, 4, 5, and 6, respectively.

Table 3. Distance between entities [km].

	S	F	W1	W2	C1	C2	AIR1	AIR2	SEA1	SEA2
S	-	272	1112	1263	1843	2395	2158	799	705	1474
F	272	-	1835	2115	1900	812	1168	984	1883	1914
W1	1112	1835	-	183	1564	136	1271	1714	2027	968
W2	1263	2115	183	-	683	2153	1236	1396	1483	1506
C1	1843	1900	1564	683	-	2141	2023	1481	1291	2071
C2	2395	812	136	2153	2141	-	38	1193	1693	131
AIR1	2158	1168	1271	1236	2023	38	-	1949	2057	558
AIR2	799	984	1714	1396	1481	1193	1949	-	689	1076
SEA1	705	1883	2027	1483	1291	1693	2057	689	-	472
SEA2	1474	1914	968	1506	2071	131	558	1076	472	-

Table 4. Handling costs at the hub terminals.

AIR1	AIR2	SEA1	SEA2
0.144479545	0.085694602	0.135877986	0.124274278

3.2 Transferred data

We complete the environmental interventions for the processes related to the storage and handling of incoming goods at the seaports/airports. For the storage process, we focus on the environmental flows during the operational phase, as the

Table 5. Working condition indexes for entities.

Entity	Working condition index
S	0.6150311
F	0.3042256
W1	0.5143863
W2	0.6266747
C1	0.5570957
C2	0.727054
AIR1	0.3544411
AIR2	0.6972989
SEA1	0.840461
SEA2	0.5519594

Table 6. Environmental impact from supplier.

Impact category	Environmental impact per raw material supplied
CC	483.2338756
OD	1.08232E-05
TA	1.905968855
FE	1.597908818
ME	0.07903494
HT	232.2245502
POF	1.295213717
PMF	1.720146171
TET	0.233408957
FET	5.425323187
MET	1026.42651
IR	30.61890951
ALO	84.48303963
ULO	10.90732898
NLT	0.07180657
MRD	244.1848156
FRD	63.45095509

infrastructure-related environmental flows (e.g. building construction) for warehousing are already accounted for in the construction process. Based on data on energy consumption (i.e. lighting and HVAC energy) in warehouses per m^2 of product ([2]), we use the UN ([3]) LCA report for electricity generation to quantify the environmental flows from storage processes. We assume that the same environmental interventions apply to the product handling of incoming goods at seaports/airports. The corresponding data is summarized in Table 7. Please note that the transferred data only encompasses the impact categories considered in [1]. The normalization factors for the environmental impact categories are considered to be the same as in [1].

4 Solutions of SCO and SCLCO

The SCO model of [1] and the proposed SCLCO model were implemented using the IBM CPLEX Python API in Python 3.7. SCLCO model implementation is available in Hülagü (2023). All experiments are conducted on a computer with an Intel(R) Core(TM) i7-1185G7, 3.00GHz processor and 16GB of RAM. We obtain results for both models in less than one second. The results are presented in Table 8 in the same reporting format as [1]. It provides an analysis of the environmental (W^{env}), economic

Table 7. Environmental impact on storage, per m^2 of product storage.

Impact category	Environmental impact per m^2 of product storage
CC	64.982
OD	0.000
TA	0.000
FE	2.946
ME	0.000
HT	0.000
POF	0.000
PMF	0.000
TET	0.000
FET	0.000
MET	0.000
IR	1.382
ALO	0.000
ULO	0.000
NLT	0.000
MRD	0.000
FRD	0.000

(W^{ecn}), and social (W^{soc}) impacts associated with the solutions obtained from the optimization of environmental (min W^{env}), economic (min W^{ecn}), and social (min W^{soc}) objectives.

Table 8. Environmental (W^{env}), economic (W^{ecn}), and social (W^{soc}) impacts associated with the solutions obtained from the optimization of environmental (min W^{env}), economic (min W^{ecn}), and social (min W^{soc}) objectives.

	W^{env}	W^{ecn}	W^{soc}
min W^{env}	3.944E+05	1.344E+06	-11.985
min W^{ecn}	6.201E+05	-7.092E+05	-11.719
min W^{soc}	4.620E+05	2.553E+08	-501.266

The scaling factors for the processes that are used in the environmental, economic, and social solutions (greater than zero) are provided in Table 9, 10, and 11.

References

1. Tautenhain CP, Barbosa-Póvoa AP, Mota B, Nascimento MC. An efficient Lagrangian-based heuristic to solve a multi-objective sustainable supply chain problem. European Journal of Operational Research. 2021;294(1):70–90. doi:10.1016/j.ejor.2021.01.008.

2. Fichtinger J, Ries JM, Grosse EH, Baker P. Assessing the environmental impact of integrated inventory and warehouse management. International Journal of Production Economics. 2015;170:717–729. doi:10.1016/j.ijpe.2015.06.025.

3. United Nations Economic Commission for Europe. Carbon neutrality in the UNECE region: integrated life-cycle assessment of electricity sources. UN; 2022.

Table 9. SCLCO solution for environmental objective: scaling factors, environmental impacts, economic impacts and social impacts.

Process	Units	s_0	s_1	s_2	Envrn. Impacts	Econ. Impacts	Social Impacts
RM1 production	unit	-	13585.6	184.3	128890.8	1907.9	0
RM2 production	unit	-	2433.0	33.0	23082.7	286.7	0
FP production with TCH1 using T2	unit	-	73261.3	993.7	222249.9	17152.8	0
FP arrival & storage at FW	unit	-	28976.3	0	0	4718.5	0
FP storage at FW	unit	-	0	28976.32	0	4289.5	0
FP handling at SEA1 using T2 from F	unit	-	30220.0	0	0	2613.1	0
FP handling at SEA1 using T2 from FW	unit	-	0	23061	0	-1601.1	0
FP Handling at SEA2 using SHP	unit	-	30220.0	23061	0	0	0
Usage at C2 using T2 from SEA2	unit	-	30220.0	23061	0	-1.52E+06	0
Usage at C1 using T2 from F	unit	-	14065.0	9850.677	0	-682741.9	0
Usage at C1 using T2 from FW	unit	-	0	5915.323	0	-159490.2	0
UFP Handling at SEA2 using T2 from C2	unit	-	0	30220	0	152404.9	0
UFP Handling at SEA1 using SHP from SEA2	unit	-	0	30220	0	0	0
FP Remanufacturing with TCH5 using T2 from SEA1	unit	-	0	6044	13038.7	917.2	0
FP Remanufacturing with TCH5 using T2 from C1	unit	-	0	2813	6068.5	70348.0	0
Land Transportation using T2	kgkm	-	2.48E+07	1.05E+08	646.1	0.1	0
Sea Transportation	kgkm	-	3.51E+06	1.67E+07	83.8	1.19E+05	0
T2 driving between (S,F)	trip	-	3	1	0	751.1	0
T2 driving between (F,SEA1)	trip	-	6	5	0	14026.3	0
T2 driving between (SEA2,C2)	trip	-	6	5	0	975.8	0
T2 driving between (F,C1)	trip	-	3	3	0	7686.5	0
T2 driving between (C2,SEA2)	trip	-	0	6	0	504.7	0
T2 driving between (SEA1,F)	trip	-	0	6	0	7255.0	0
T2 driving between (C1,F)	trip	-	0	3	0	3660.2	0
Constructing/Maintaining F	m^2	145.5	-	-	297.9	2.91E+06	-0.4
Planning T2 at S	truck	1	-	-	0	16592.1	-0.6
Planning T2 at F	truck	1	-	-	0	15287.7	-0.3
Planning T2 at C1	truck	1	-	-	0	18654.2	-0.5
Planning T2 at SEA2	truck	1	-	-	0	27479.0	-0.5
Planning T2 at C2	truck	1	-	-	0	25540.0	-1.9
Planning T2 at SEA1	truck	1	-	-	0	18872.9	-1.2
Installing TCH1	unit	1	-	-	0	112935.2	-3.0
Installing TCH5	unit	1	-	-	0	112263.9	-3.0
Operating S	unit	1	-	-	0	0	0
Operating F	unit	1	-	-	0	21679.4	-0.6
Operating SEA1	unit	1	-	-	0	10031.2	0
Operating SEA2	unit	1	-	-	0	9862.4	0

Table 10. SCLCO solution for economic objective: scaling factors, environmental impacts, economic impacts and social impacts.

Process	Units	s_0	s_1	s_2	Envrn. Impacts	Econ. Impacts	Social Impacts
RM1 production	unit	-	13045.8	10097.2	216626.8	3083.2	0
RM2 production	unit	-	8513.9	6589.5	141373.5	1688.1	0
FP production with TCH2 using T1	unit	-	46103.2	35682.8	258786.9	12563.8	0
FP arrival & storage at FW	unit	-	32038.2	21242.8	0	8361.8	0
FP storage at FW	unit	-	0	1818.2	0	269.2	0
FP Handling at SEA1 using T2 from FW	unit	-	30220.0	23061.0	0	-3909.0	0
FP Handling at SEA2 using SHP	unit	-	30220.0	23061.0	0	0	0
Usage at C2 using T1 from SEA2	unit	-	30220.0	23061.0	0	-1.52E+06	0
Usage at C1 using T2 from F	unit	-	14065.0	15766.0	0	-8.42E+05	0
UFP Handling at SEA2 using T1 from C2	unit	-	0	4524.2	0	22816.5	0
UFP Handling at SEA1 using SHP from SEA2	unit	-	0	4524.2	0	0	0
FP Remufacturing with TCH5 using T1 from SEA1	unit	-	0	904.8	1952.0	137.3	0
FP Rem. with TCH5 using T1 from C1	unit	-	0	421.1	908.5	10531.8	0
Land Transportation using T1	kgkm	-	4.9E+06	1.7E+07	122.2	0	0
Land Transportation using T2	kgkm	-	2.1E+07	1.8E+07	192.2	0	0
Sea Transportation	kgkm	-	3.5E+06	4.8E+06	34.3	49953.8	0
T1 driving between (S,F)	trip	-	5.0	4.0	0	1811.8	0
T2 driving between (F,SEA1)	trip	-	6.0	5.0	0	14026.3	0
T1 driving between (SEA2,C2)	trip	-	6.0	5.0	0	1065.5	0
T2 driving between (F,C1)	trip	-	3.0	3.0	0	7686.5	0
T1 driving between (C2,SEA2)	trip	-	0	1.0	0	91.9	0
T1 driving between (SEA1,F)	trip	-	0	1.0	0	1320.3	0
T1 driving between (C1,F)	trip	-	0	1.0	0	1332.2	0
Constructing/Maintaining F	m^2	57	-	-	116.7	1.1E+06	-0.2
Planning T1 at S	truck	1	-	-	0	14894.5	-0.6
Planning T2 at F	truck	1	-	-	0	15287.7	-0.3
Planning T1 at C1	truck	1	-	-	0	16956.5	-0.5
Planning T1 at SEA2	truck	1	-	-	0	25781.3	-0.5
Planning T1 at C2	truck	1	-	-	0	23842.3	-1.9
Planning T1 at SEA1	truck	1	-	-	0	17175.3	-1.2
Installing TCH2	unit	1	-	-	0	1.11E+05	-3.0
Installing TCH5	unit	1	-	-	0	112263.9	-3.0
Operating S	unit	1	-	-	0	0	0
Operating F	unit	1	-	-	0	21679.4	-0.6
Operating SEA1	unit	1	-	-	0	10031.2	0
Operating SEA2	unit	1	-	-	0	9862.4	0

Table 11. SCLCO solution for social objective: scaling factors, environmental impacts, economic impacts and social impacts.

Process	Units	s_0	s_1	s_2	Envrn. Impacts	Econ. Impacts	Social Impacts
RM1 production	unit	-	12621.6	2845.7	144779.9	2109.9	0
RM2 production	unit	-	2260.4	509.6	25928.3	317.0	0
FP production with TCH1 using T1	unit	-	68063.0	15345.9	249648.0	18968.1	0
FP arrival & storage at W1 using T1	unit	-	3578.0	1.0	0	582.8	0
FP storage at W1	unit	-	0	3577.0	0	529.5	0
FP handling at AIR1 using T1 from F	unit	-	50420.0	0	0	4635.7	0
FP Handling at AIR1 using T1 from W1	unit	-	1.0	3244.0	0	271.2	0
FP Handling at AIR2 using PLN	unit	-	50421.0	3244.0	0	0.0	0
FP arrival & storage at W2 using T1 from AIR2	unit	-	20202.0	3244.0	0	3769.9	0
FP storage at W2	unit	-	0	20201.0	0	2990.5	0
Usage at C2 using T1 from W2	unit	-	1.0	23061.0	0	-621805.2	0
Usage at C2 using T1 from AIR2	unit	-	30219.0	0	0	-896248.2	0
Usage at C1 using T1 from F	unit	-	14065.0	15766.0	0	-842232.1	0
UFP arrival & storage at W2 using T1 from C2	unit	-	0	30220.0	0	154705.8	0
FP Remanufacturing with TCH4 using T1 from C1	unit	-	0	421.1	1209.3	10535.8	0
Land Transportation using T1	kgkm	-	4.1E+07	9.0E+07	748.6	0.1	0
Air Transportation	kgkm	-	2.4E+07	1.6E+06	137.2	651052.5	0
T1 driving between (S,F)	trip	-	3.0	1.0	0	820.1	0
T1 driving between (F,W1)	trip	-	1.0	1.0	0	2701.9	0
T1 driving between (F,AIR1)	trip	-	10.0	0	0	9008.5	0
T1 driving between (W1,AIR1)	trip	-	1.0	1.0	0	1871.5	0
T1 driving between (AIR2,W2)	trip	-	4.0	1.0	0	5285.6	0
T1 driving between (W2,C2)	trip	-	1.0	5.0	0	9208.5	0
T1 driving between (AIR2,C2)	trip	-	6.0	0	0	5520.8	0
T1 driving between (F,C1)	trip	-	3.0	4.0	0	9725.0	0
T1 driving between (C2,W2)	trip	-	0	6.0	0	9057.5	0
T1 driving between (C1,F)	trip	-	0	1.0	0	1332.2	0
Constructing/Maintaining F	m^2	11016	-	-	2.3E+04	2.2E+08	-33.1
Constructing/Maintaining W1	m^2	4109	-	-	8413.1	1.5E+07	-26.0
Constructing/Maintaining W2	m^2	4187	-	-	8572.8	1.6E+07	-22.1
Planning T1 at F	truck	1	-	-	0	13590.0	-0.3
Planning T1 at S	truck	1	-	-	0	14894.5	-0.6
Planning T1 at W1	truck	1	-	-	0	13081.1	-0.6
Planning T1 at C1	truck	1	-	-	0	16956.5	-0.5
Planning T1 at AIR2	truck	1	-	-	0	13171.4	-1.1
Planning T1 at W2	truck	1	-	-	0	7983.6	-0.5
Planning T1 at C2	truck	212	-	-	0	5054578.1	-408.0
Installing TCH1	unit	1	-	-	0	112935.2	-3.0
Installing TCH4	unit	1	-	-	0	114014.1	-3.0
Operating S	unit	1	-	-	0	0.0	0
Operating F	unit	1	-	-	0	21679.4	-0.6
Operating W1	unit	1	-	-	0	21679.4	-1.3
Operating W2	unit	1	-	-	0	10839.7	-0.5
Operating AIR1	unit	1	-	-	0	12092.9	0
Operating AIR2	unit	1	-	-	0	14916.2	0
